# The Application of Fluorescence In Situ Hybridization in the Prescreening of *Veronica* Hybrids

**DOI:** 10.3390/plants13091264

**Published:** 2024-05-01

**Authors:** Hye-Wan Park, Samantha Serafin Sevilleno, My Khanh Tran Thi Ha, Raisa Aone Cabahug-Braza, Ji-Hun Yi, Ki-Byung Lim, Wonwoo Cho, Yoon-Jung Hwang

**Affiliations:** 1 Department of Convergence Science, Sahmyook University, Seoul 01795, Republic of Korea; hyewan0204@gmail.com (H.-W.P.); samanthasevilleno20@gmail.com (S.S.S.); 2 Institute for Global Health Innovations, Duy Tan University, Danang 550000, Vietnam; hattmykhanh@duytan.edu.vn; 3 Plant Genetics and Breeding Institute, Sahmyook University, Seoul 01795, Republic of Korea; raisaaone@gmail.com; 4 Division of Garden and Plant Resources, Korea National Arboretum, Pocheon 11186, Republic of Korea; easy2641@korea.kr; 5 Department of Horticultural Science, Kyungpook National University, Daegu 41566, Republic of Korea; kblim@knu.ac.kr

**Keywords:** *Veronica*, FISH, cytogenetics, rDNA, marker-assisted breeding

## Abstract

Fluorescence in situ hybridization (FISH), a molecular cytogenetic technique that enables the visualization and identification of specific DNA sequences within chromosomes, has emerged as a pivotal tool in plant breeding programs, particularly in the case of *Veronica* species. *Veronica*, a genus with a complex reproductive system, often poses challenges in accurately identifying hybrids because of its tendency to hybridize, which leads to intricate genetic variation. This study focused on the use of FISH as a prescreening method to identify true hybrids in *Veronica* breeding programs. FISH analysis was first performed on the parents to identify their 45S and 5S rDNA signals, along with their respective chromosome numbers. The signals were then compared with those of the twenty progenies with reference to their supposed parents. Five true hybrids, seven self-pollinated progenies, and eight false hybrids were identified through FISH. The findings highlight the significance of FISH as a screening method that contributes significantly to the efficiency of *Veronica* breeding programs by ensuring the preservation of desired genetic traits and minimizing the inadvertent inclusion of misidentified hybrids. To conclude, this study underscores the vital role of FISH in enhancing the precision and success of breeding programs and opens new avenues for improved breeding strategies and crop development.

## 1. Introduction

Fluorescence in situ hybridization (FISH) is an important molecular cytogenetic tool widely used to distinguish complementary DNA sequences using fluorescently labeled probes [1,2]. Along with the chromosome karyotype, FISH data provide the basic information needed for successful crossbreeding, which includes ploidy level, species chromosome characteristics, and parental origin [3]. FISH analysis helps determine the ploidy level and detect aneuploidy, which are important factors affecting pollen fertility and crossing efficiency in roses [4]. Likewise, results from this technique make it easier to visually authenticate hybrids and help track the origins of specific chromosomes, as applied in lily breeding [5]. Owing to its efficient detection and accuracy, the use of FISH analysis for ornamental plants, which have high demand and ornamental significance, is invaluable.

Currently, interest in the use of *Veronica* species as indoor and landscape plants is at a peak. *Veronica*, a large genus of the Plantaginaceae family with over 450 species, is highly adaptable to various ecological conditions and horticulturally valued for its prolonged flowering and easy maintenance. Their long flowering period varies from spring to fall, and they come in a wide array of colors, including white, pink, purple, and blue [6,7]. Between 2018 and 2019, there was an eight-fold increase in the average trading volume of *Veronica* flowers and an almost 50% increase in its unit price [8]. Owing to its desirable ornamental characteristics and increased market demand, an increasing number of studies have been conducted on its classification, morphology, propagation, and breeding [9,10,11].

Several domestic and wild *Veronica* species have been used as parents for plant breeding programs for *Veronica* species with desirable characteristics. In Korea, certain native taxa have been evaluated for their growth and flowering characteristics and have at least 40 to over 110 spikes per plant with minute florets ranging from 7 to 10 mm in diameter [11]. Because *Veronica* is self-compatible, many progenies are produced in one crossing and have a mixture of inbred, false, and true hybrids; thus, self-pollination is a possibility and inevitable [12,13]. A similar situation was observed in closely comparable spiked flowers of *Plantago* species [14].

Although there are traditional screening methods, such as direct investigation using gross morphological or physical characteristics, there are some cases where these methods may not be sufficient, as hybrids may resemble both parents [15]. In breeding programs, successful interspecific hybridization has been screened using traditional approaches coupled with high-throughput methods such as flow cytometry [16] and single-nucleotide polymorphism markers [17]. However, despite their advantages, these methods can only provide limited information for visibly identifying chromosome structure and organization. An alternative screening method that provides essential information for interspecific hybrids is the use of molecular cytogenetic tools such as karyotyping and FISH [18].

FISH is a well-established molecular cytogenetic technique that primarily focuses on chromosomal-level phylogeny investigation [19]. FISH not only locates a target sequence but also enables qualitative and quantitative analysis by employing labeled nucleic acid probes in conjunction with chromosomes, interphase nuclei, or DNA fibers [20]. This technique has found extensive applications in the identification of specific chromosomal regions and analysis of their composition, spatial positioning, and dynamic changes in chromatin during the cell cycle [19,20,21]. Moreover, FISH has been widely employed to elucidate the physical map, structure, and evolution of the genome and study interspecies relationships [3,5,20]. Ribosomal DNA (rDNA), comprised of 45S and 5S, has been shown to be the most widely used markers, which present a high copy number and are tandemly arranged with different chromosomal distributions [21,22].

FISH analysis was conducted to determine the cytogenetic features of native Korean *Veronica* taxa, including the chromosome number and rDNA distribution patterns [23]. To date, FISH data have been used to cytogenetically characterize wild species and cultivars to support evolutionary and phylogenetic studies of *Veronica* spp. [13,24]. We hypothesized that the identification of true hybrids might be based on rDNA distribution patterns that are accurately and visibly identifiable in chromosome spreads. By determining the 5S and 45S loci of the parents and progenies, we were able to differentiate true hybrids from self-pollination and false offspring produced via breeding programs. Only a limited number of studies have used rDNA-FISH analysis as a screening method; hence, we conducted this study.

## 2. Results

To determine the true hybrids, FISH analysis was first performed on the parents to identify their 45S and 5S rDNA signals, along with their respective chromosome numbers. The signals were then compared with those of the 20 progenies with reference to their supposed parents. The FISH results for the parents as well as the results of crossing studies producing true, self-pollinated, and false hybrids are presented in Table 1.

### 2.1. Parents

**Figure 1:**
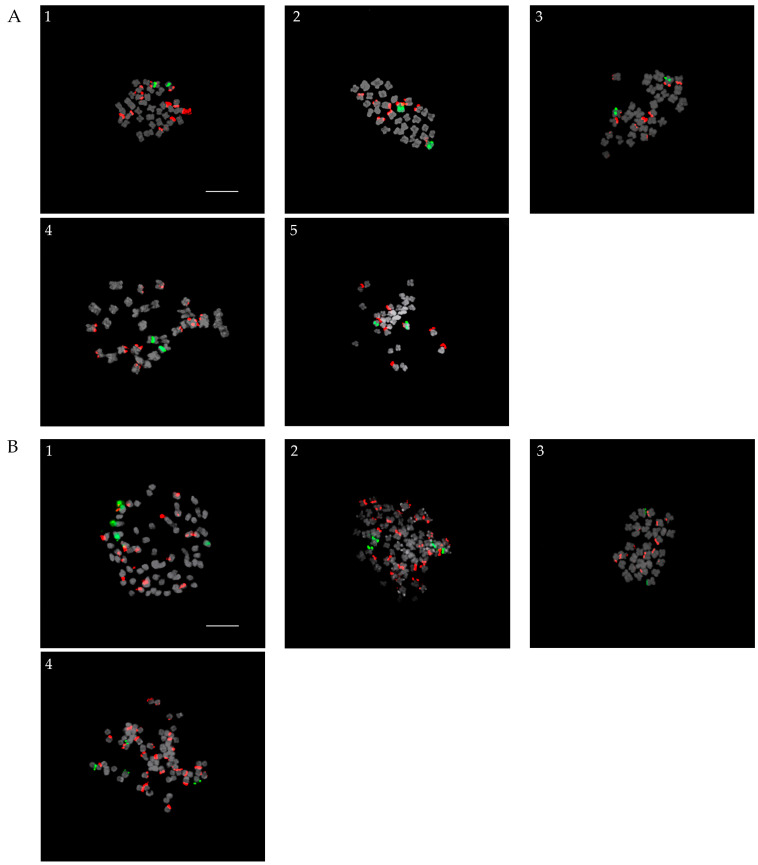
Parent *Veronica* species. (**A**) Korean native species, namely: (**1**) *V. pyrethrina* Nakai; (**2**) V. *dahurica* Steven; (**3**) *V. pusanensis* Y. N. Lee; (**4**) *V. kiusiana* var. glabrifolia; (**5**) *V. nakaiana* Ohwi and (**B**) cultivars, namely: (**1**) *V. spicata* ‘Ulster Blue Dwarf’; (**2**) *V. longifolia* ‘Blue Shades’; (**3**) *V. spicata* f. nana ‘Blauteppich’; and (**4**) *V.* ‘Veronica Blue’. 5S and 45S rDNA signals are indicated by the green and red fluorescence, respectively. Scale bar = 10 µm, 400× magnification.

There were nine parents of the screened progenies, of which five were native Korean species (Figure 1A) and four were cultivars (Figure 1B). All Korean native species as well as one cultivar, *Veronica spicata* f. nana ‘Blauteppich’, showed diploid complements 2*n* = 34, while the other three cultivars were tetraploids with 2*n* = 68 (Figure 1). FISH analysis revealed that diploid species and cultivars possessed a pair of 5S rDNA loci, whereas tetraploid parents possessed two pairs of 5S rDNA loci. In contrast, a variable number of 45S rDNA loci were observed in these species and cultivars. Four pairs of 45S rDNA loci were identified in *Veronica nakaiana* Ohwi. *Veronica dahurica* Steven and *Veronica pusanensis* Y. N. Lee possessed five pairs of 45S rDNA loci, whereas *Veronica pyrethrina* Nakai, *Veronica kiusiana* var. glabrifolia, and *V. spicata* f. nana ‘Blauteppich’ had six pairs. The tetraploid cultivars, *Veronica spicata* ‘Ulster Blue Dwarf’, and *Veronica* ‘Veronica Blue’, as well as *Veronica longifolia* ‘Blue Shades’, were observed to have nine and fourteen pairs of 45S rDNA loci, respectively.

### 2.2. True Hybrids

**Figure 2:**
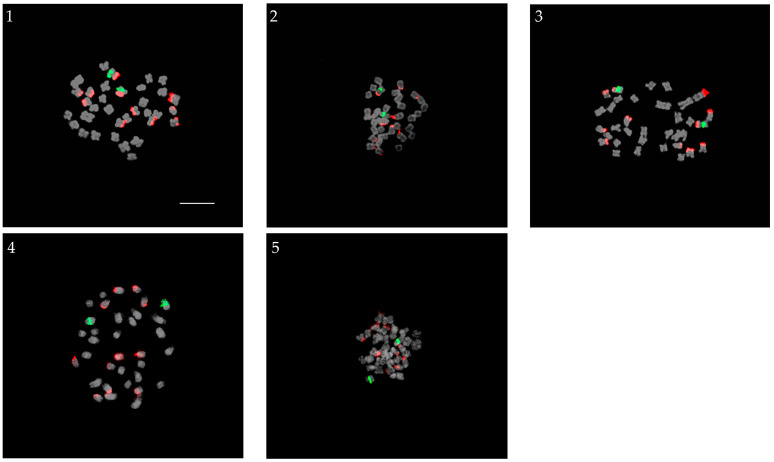
Progenies screened as true hybrids: (**1**) Ve-31 (*V. pyrethrina* Nakai × *V. pusanensis* Y. N. Lee); (**2**) Ve-55-1 (*V. pusanensis* Y. N. Lee × *V. pyrethrina* Nakai); (**3**) Ve-57-1 (*V. pusanensis* Y. N. Lee × *V. kiusiana* var. glabrifolia); (**4**) Ve-86 (*V. kiusiana* var. glabrifolia × *V. dahurica* Steven); and (**5**) Ve-88 (*V. kiusiana* var. glabrifolia × *V. pusanensis* Y. N. Lee). 5S and 45S rDNA signals are indicated by the green and red fluorescence, respectively. Scale bar = 10 µm, 400× magnification.

The results of the hybridization studies, which produced five true hybrids, are shown in Figure 2. The parents used for crossing were all diploid, with one pair of 5S rDNA loci and either five or six pairs of 45S rDNA loci (Figure 2). The results showed that all five progenies had diploid complements with 2*n* = 34, similar to their parents. A pair of 5S rDNA loci and 11 45S rDNA FISH signals was detected in all hybrids. The FISH signals of 5S and 45S rDNA in the chromosomal complement corresponded to the average number of rDNA signals in both parents. The female parents of Ve-31, Ve-86, and Ve-88 possessed six pairs of 45S rDNA loci, whereas the male parents had five pairs of 45S rDNA loci. In contrast, Ve-55-1 and Ve 57-1 whose female parent is *V. pusanensis* Y. N. Lee, contain five pairs of 45S rDNA loci, while the male parents both have six pairs of 45S rDNA FISH signals. All progenies had the expected number of eleven 45S rDNA sites.

### 2.3. Self-Pollinated Progenies

**Figure 3:**
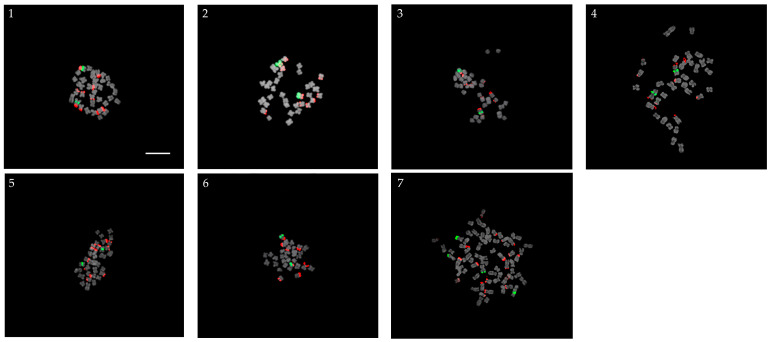
Progenies screened as self-pollinated: (**1**) Ve-48 (*V. pusanensis* Y. N. Lee × *V. longifolia* ‘Blue Shades’); (**2**) Ve-56 (*V. pusanensis* Y. N. Lee × *V. kiusiana* var. glabrifolia (S2)); (**3**) Ve-59-2 (*V. dahurica* Steven × *V. spicata* f. nana ‘Blauteppich’); (**4**) Ve-65-2 (*V. dahurica* Steven × *V. pyrethrina* Nakai); (**5**) Ve-77 (*V. kiusiana* var. glabrifolia × *V. spicata* f. nana ‘Blauteppich’); (**6**) Ve-91 (*V. nakaiana* Ohwi × *V. spicata* f. nana ‘Blauteppich’); and (**7**) Ve-153 (*V. spicata* ‘Ulster Blue Dwarf’ × *V. kiusiana* var. glabrifolia). 5S and 45S rDNA signals are indicated by the green and red fluorescence, respectively. Scale bar = 10 µm, 400× magnification.

The seven crossing studies that resulted in the self-pollinated *Veronica* species are shown in Figure 3. All self-pollinated progenies were diploids (2*n* = 34), possessing one pair of 5S rDNA loci, except for Ve-153, which was tetraploid (2*n* = 68) with two pairs of 5S rDNA loci (Figure 3). Ve-48 and Ve-56, whose female parent was *V. pusanensis* Y. N. Lee, possessed five pairs of 45S rDNA loci. Ve-59-2 and Ve-65-2, with *V. dahurica* Steven as their female parent, also had five pairs of 45S rDNA loci. Ve-77 had a similar number as its parents, with six pairs of 45S rDNA loci. Ve-91 had four pairs of 45S rDNA loci, similar to its female parent, *V. nakaiana* Ohwi. Nine pairs of 45S rDNA signals were detected in Ve-153, similar to its female parent, *V.* ‘Ulster Blue Dwarf’.

### 2.4. False Hybrids

**Figure 4:**
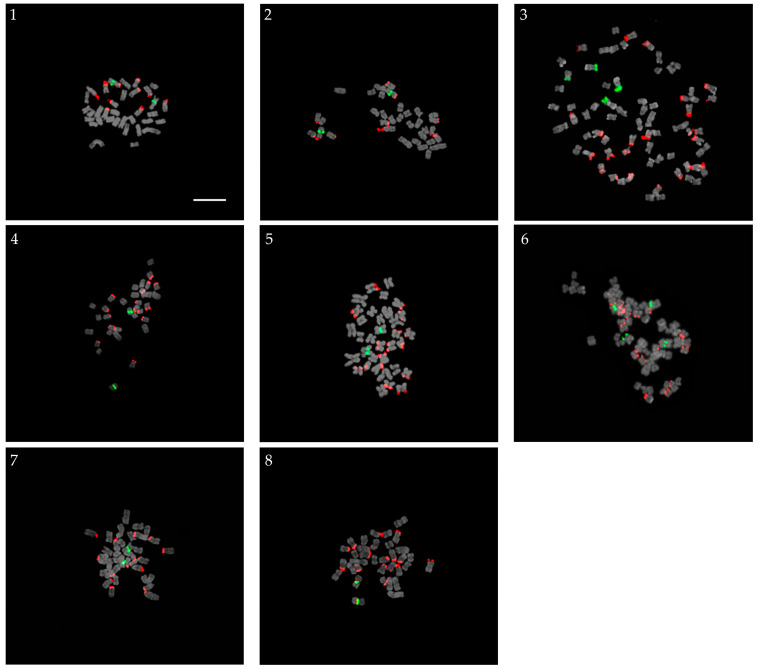
Progenies screened as false hybrids: (**1**) Ve-33 (*V. pyrethrina* Nakai × *V. spicata* f. nana ‘Blauteppich’); (**2**) Ve-38 (*V. pyrethrina* Nakai × *V. spicata* ‘Ulster Blue Dwarf’); (**3**) Ve-65-1 (*V. dahurica* Steven × *V. pyrethrina* Nakai); (**4**) Ve-75 (*V. kiusiana* var. glabrifolia × *V. spicata* f. nana ‘Blauteppich; (**5**) Ve-87 (*V. kiusiana* var. glabrifolia × *V. pusanensis* Y. N. Lee); (**6**) Ve-109 (*V.* ‘Veronica Blue’ × *V. kiusiana* var. glabrifolia); (**7**) Ve-131 (*V. spicata* f. nana ‘Blauteppich’ × *V. pyrethrina* Nakai); and (**8**) Ve-132 (*V. spicata* f. nana ‘Blauteppich’ × *V. pyrethrina* Nakai). 5S and 45S rDNA signals are indicated by the green and red fluorescence, respectively. Scale bar = 10 µm, 400× magnification.

Eight false hybrids were produced in crossing studies, as shown in Figure 4. Progenies are hypothesized to be false hybrids when their chromosome number or rDNA loci is different from the average chromosome number and rDNA loci of both parents or different from the corresponding number of chromosomes or rDNA loci in one of their parents. All progenies were diploid (2*n* = 34), except for Ve-65-1 and Ve-109, which were tetraploid (2*n* = 68) (Figure 4). All diploid progenies possessed one pair of 5S rDNA loci, whereas tetraploid progenies had two pairs of 5S rDNA loci. The number of 45S rDNA sequences varied among the progenies. Ve-87, whose supposed parents were similar to the true hybrid Ve-88, was observed to have 16 45S rDNA sites in contrast to the 11 FISH signals found in Ve-88. Eight pairs of 45S hybridization signals were detected in Ve-75, with the same parent as the self-pollinated progeny, Ve-77, which possessed only six pairs of 45S rDNA signals. Ve-65-1 and Ve-65-2 (self-pollinated progeny) had the same parents; however, the former was found to be tetraploid with ten pairs of 45S rDNA loci compared to the latter, which was diploid with only five pairs of 45S rDNA loci. Ve-131 and Ve-132 had the same parents but possessed different 45S rDNA loci at 16 and 15, respectively. Ve-38 and Ve-109, which resulted from a supposed cross-hybridization between a diploid and a tetraploid parent, did not possess the expected triploid chromosome number but were diploid and tetraploid, respectively, and had different 45S rDNA signals in contrast with their supposed parents. 

## 3. Discussion

In plant breeding programs, new experimental hybrids are produced by crossing two parent crops. In many plant species such as lilies [25], crossbreeding is a fundamental breeding strategy in which hybrid affinity is crucial for success. The production of false hybrids is a frequent outcome of hybridization, making it difficult to determine the authenticity of the hybrid [26]. Following cross-hybridization, distinguishing true hybrids is not only vital in developing a breeding program but also essential in enhancing hybrid varieties. However, current approaches to hybrid identification, whether through morphological or molecular means, are both laborious and costly and require specialized knowledge, skills, and specific laboratory equipment [27].

Since the number of chromosomes and karyotypes of a species or cultivar is generally stable, the conventional cytogenetic method of karyotype analysis has been useful in rapidly classifying plant species and verifying alien disomic addition lines in breeding through the identification of some basic cytological parameters [28,29,30]. However, it cannot distinguish individual chromosomes with similar morphology, size, and parental origin from a pair of chromosomes, such as *Veronica* species with small and similarly sized chromosomes [6]. FISH, which maps repetitive or single-copy sequences on the chromosomes, serves as an important tool in plant cytogenetics. 5S and 45S rDNA, which are highly conserved tandem repeat sequences, are generally used as cytogenetic markers for routine FISH analyses of plant species [31,32,33]. These genetic markers display variability in both intraspecific and interspecific hybrids, as indicated by differences in the intensity, number, and location of hybridization signals [34]. Hybrids can be confirmed cytogenetically based on chromosomal traits using in situ hybridization techniques at the earliest growth stage. FISH is instrumental in detecting hybrids with probes employed as markers to determine the number and position of rRNA genes on chromosomes [35].

In *Veronica* progenies screened as true hybrids, the FISH signals of 5S and 45S rDNA in their chromosome complements were the average for both parents. FISH has also been utilized to validate hybridization status and investigate genetic diversity in progenies or developed hybrids in other important horticultural plants, such as sweet potato [36], *Passiflora* [37], and *Thinopyrum elongatum* [38]. The first attempt to use FISH with 25S rDNA as a probe proved to be sufficient for hybrid status verification in some *Lilium* hybrids [35]. In a study by Wang et al. [5], Asiatic hybrid lilies with different ploidy levels were crossbred. Consequently, FISH analysis was successfully conducted on the offspring using 45S rDNA as a probe to visually confirm the authenticity of the hybrid and trace the origins of specific chromosomes. In addition, FISH karyotype analyses using 5S and 45S rDNA probes in *xBrassicaraphanus* line BB#5, a hybrid of *Brassica rapa* and *Raphanus sativus*, showed a combined rDNA pattern for the two parental species [33]. In first-generation (F1) hybrids, the 45S rDNA loci inherited from each parent are often conserved and undergo differential transcriptional silencing [39,40,41].

FISH analyses of *Veronica* hybrids screened as self-pollinated showed similar chromosome numbers as the female parents, as well as their 5S and 45S rDNA FISH signals. Ve-56 had parents similar to the true hybrid Ve-57-1; however, the number of 45S rDNA sites detected was not the expected average of both parents but was comparable to that of the female parent, *V. pusanensis* Y. N. Lee, and Ve-48. Hybridization between the tetraploid *V.* ‘Ulster Blue Dwarf’ and the diploid *V. kiusiana* var. glabrifolia resulted in a tetraploid progeny (Ve-153) with rDNA signals similar to those of the female parent instead of the expected triploid hybrid with 2*n* = 51. Ve-59-2 and Ve-65-2 had similar numbers of hybridization signals as their female parent, *V. dahurica* Steven. Ve-91, a cross-hybridization between *V. nakaiana* Ohwi and *V. spicata* f. nana ‘Blauteppich’, had eight 45S rDNA FISH signals similar to those of its female parent. Some *Veronica* species, such as *Veronica cymbalaria* Bodard and *Veronica persica* Poir, have automatic self-crossing abilities [42]. Furthermore, *V. spicata* subspecies were described as self-compatible, protogynous, and entomophilous species, and were found to be self-pollinated when grown in strict isolation [43]. *Veronica* is self-compatible; thus, self-pollination during cross-hybridization is possible [12,13]. In tomatoes, less hybridization occurs because of the large number of self-pollinating plants [44].

False hybrids may arise from misclassification or misidentification of parent plants. Eight false or misidentified hybrids were classified using rDNA-FISH analysis, three of which had parents similar to the identified true and self-pollinated *Veronica* species; however, their chromosomal characteristics were different. The 45S rDNA signal distribution of these hybrids did not correspond to the average rDNA loci of both parents or at least to the rDNA loci similar to one of their parents, thus characterizing them as false hybrids.

In interspecific hybrid breeding programs, a reliable method for identifying parental species and verifying true hybrids is necessary to ensure the production of desired materials and achieve the expected genetic gains [45]. FISH using 5S and 45S rDNA probes in metaphase chromosomes, as confirmed in this study, allows for a quick and clear determination of the genomic composition of hybrid plants [46,47], making it a suitable tool to identify hybrids in breeding programs and to analyze the genomes of the genus *Veronica.*

## 4. Materials and Methods

### 4.1. Plant Materials

Young roots were harvested from the plant materials of the Korean *Veronica* wild species and the cultivars that served as parents (Table 2).

Similarly, twenty (20) progenies (Table 3) were obtained from the Useful Plant Resources Center of the Korea National Arboretum, Republic of Korea.

### 4.2. Chromosome Preparation

Modified fixation and chromosome preparation were performed following the methods described by Ha et al. [23]. Properly cleaned and washed harvested root tips were treated with 2 mM 8-hydroxyquinoline for 5 h at 18 °C. Carnoy’s solution (3:1, acetic acid: ethanol, *v*/*v*) was used to fix the roots overnight at 25 °C. The root tips were then transferred to a 70% ethanol solution at 4 °C to preserve the materials.

The fixed root tips were placed in distilled water prior to enzyme mixture (1% cellulose, cytohelicase, and pectolyase) treatment at 37 °C for 90 min. The enzyme-treated roots were transferred to a 1.5 mL tube containing Carnoy’s solution and vortexed for 20 s. Homogenized root meristems were placed on ice for 5 min and centrifuged at 13,000 rpm to collect the pellets. The supernatant was discarded, and the pellet was immediately resuspended in an acetic acid–ethanol (9:1) solution. The final suspension was pipetted on an 80 °C pre-warmed glass slide in a humid chamber and air-dried at room temperature.

### 4.3. Fluorescence In Situ Hybridization

FISH was performed using the modified procedure described by Lim et al. [48]. Pre-labeled probes (PLOPs) of 5S and 45S rDNA sequences were used following the methods described by Waminal et al. [49]. The FISH hybridization mixture consists of 50% formamide, 10% dextran sulfate, 20× saline sodium citrate (SSC) buffer, 50 ng/μL of each PLOP, and nuclease-free water with a 40 μL total volume. The mixture was spread onto prepared chromosomal slides and denatured on a slide heater at 80 °C for 5 min. The slides were then incubated at room temperature in a humid chamber for 30 min. After hybridization, the slides were washed sequentially with 2× SSC at room temperature for 10 min, 0.1× SSC at 42 °C for 25 min, and 2× SSC at room temperature for 5 min and then dehydrated in a series of ethanol concentrations of 70%, 90%, and 100% at room temperature for 3 min each. The slides were counterstained with Vectashield (H-1000, Vector Laboratories, Newark, CA, USA) with 1 µg/mL^−1^ 4′,6-diamidino-2-phenylindole (Roche, Indianapolis, IN, USA) and observed under a fluorescence microscope (Olympus BX53, Olympus, Tokyo, Japan) with a built-in CCD camera (CoolSNAP™ cf, Photometrics, Tucson, AZ, USA) using an oil lens (×100 magnification).

## 5. Conclusions

The study results demonstrated the effectiveness of FISH in distinguishing true hybrids from plants produced through other genetic mechanisms. These findings highlight the critical role of FISH as a rapid prescreening method that greatly enhances the efficiency of *Veronica* breeding programs by maintaining the desired genetic characteristics and reducing the inconvenience and consequences of incorporating misidentified hybrids.

## Figures and Tables

**Table 1:** FISH analyses of parent Korean native *Veronica* species and cultivars, and the results of crossing studies producing true hybrids, self-pollinated *Veronica* species, and false *Veronica* hybrids.

Plant Sample		Species/Cultivars	Chromosome Number (2*n*)	FISH Signals	
				5S rDNA	45S rDNA
*Parents*		*Veronica pyrethrina* Nakai	34	2	12
		*Veronica dahurica* Steven	34	2	10
		*Veronica pusanensis* Y. N. Lee	34	2	10
		*Veronica kiusiana* var. glabrifolia	34	2	12
		*Veronica nakaiana* Ohwi	34	2	8
		*Veronica spicata* ‘Ulster Blue Dwarf’	68	4	18
		*Veronica longifolia* ‘Blue Shades’	68	4	28
		*Veronica spicata* f. nana ‘Blauteppich’	34	2	12
		*Veronica* ‘Veronica Blue’	68	4	28
**Classification**	**Code**	**Supposed Parents (Mo × Fa)**	**Chromosome Number (2*n*)**	**FISH Signals**	
				**5S rDNA**	**45S rDNA**
*True hybrids*	Ve-31	*V. pyrethrina* Nakai × *V. pusanensis* Y. N. Lee	34	2	11
	Ve-55-1	*V. pusanensis* Y. N. Lee × *V. pyrethrina* Nakai	34	2	11
	Ve-57-1	*V. pusanensis* Y. N. Lee × *V. kiusiana* var. glabrifolia	34	2	11
	Ve-86	*V. kiusiana* var. glabrifolia × *V. dahurica* Steven	34	2	11
	Ve-88	*V. kiusiana* var. glabrifolia × *V. pusanensis* Y. N. Lee	34	2	11
*Self-pollinated progenies*	Ve-48	*V. pusanensis* Y. N. Lee × *V. longifolia* ‘Blue Shades’	34	2	10
	Ve-56	*V. pusanensis* Y. N. Lee × *V. kiusiana* var. glabrifolia (S2)	34	2	10
	Ve-59-2	*V. dahurica* Steven × *V. spicata* f.nana ‘Blauteppich’	34	2	10
	Ve-65-2	*V. dahurica* Steven × *V. pyrethrina* Nakai	34	2	10
	Ve-77	*V. kiusiana* var. glabrifolia × *V. spicata* f. nana ‘Blauteppich’	34	2	12
	Ve-91	*V. nakaiana* Ohwi × *V. spicata* f. nana ‘Blauteppich’	34	2	8
	Ve-153	*V.* ‘Ulster Blue Dwarf’ × *V. kiusiana* var. glabrifolia	68	4	18
*False hybrids*	Ve-33	*V. pyrethrina* Nakai × *V. spicata* f. nana ‘Blauteppich’	34	2	10
	Ve-38	*V. pyrethrina* Nakai × *V. spicata* ‘Ulster Blue Dwarf’	34	2	10
	Ve-65-1	*V. dahurica* Steven × *V. pyrethrina* Nakai	68	4	20
	Ve-75	*V. kiusiana* var. glabrifolia × *V. spicata* f. nana ‘Blauteppich’	34	2	16
	Ve-87	*V. kiusiana* var. glabrifolia × *V. pusanensis* Y. N. Lee	34	2	16
	Ve-109	*V.* ‘Veronica Blue’ × *V. kiusiana* var. glabrifolia	68	4	16
	Ve-131	*V. spicata* f. nana ‘Blauteppich’ × *V. pyrethrina* Nakai	34	2	16
	Ve-132	*V. spicata* f. nana ‘Blauteppich’ × *V. pyrethrina* Nakai	34	2	15

**Table 2:** *Veronica* taxa used as parents in the breeding program.

No.	*Veronica* Taxa
Korean native species	
1	*Veronica pyrethrina* Nakai
2	*Veronica dahurica* Steven
3	*Veronica pusanensis* Y. N. Lee
4	*Veronica kiusiana* var. glabrifolia
5	*Veronica nakaiana* Ohwi
Cultivars	
1	*Veronica spicata* ‘Ulster Blue Dwarf’
2	*Veronica longifolia* ‘Blue Shades’
3	*Veronica spicata* f. nana ‘Blauteppich’
4	*Veronica* ‘Veronica Blue’

**Table 3:** Progenies developed from cross breeding with their respective population code.

No.	Code	Supposed Parents (Mo × Fa)
1	Ve-31	*V. pyrethrina* Nakai × *V. pusanensis* Y. N. Lee
2	Ve-33	*V. pyrethrina* Nakai ×*V. spicata* f. nana ‘Blauteppich’
3	Ve-38	*V. pyrethrina* Nakai × *V. spicata* ‘Ulster Blue Dwarf’
4	Ve-48	*V. pusanensis* Y. N. Lee × *V. longifolia* ‘Blue Shades’
5	Ve-55-1	*V. pusanensis* Y. N. Lee × *V. pyrethrina* Nakai
6	Ve-56	*V. pusanensis* Y. N. Lee × *V. kiusiana* var. glabrifolia (S2)
7	Ve-57-1	*V. pusanensis* Y. N. Lee × *V. kiusiana* var. glabrifolia
8	Ve-59-2	*V. dahurica* Steven × *V. spicata* f. nana ‘Blauteppich’
9	Ve-65-1	*V. dahurica* Steven × *V. pyrethrina* Nakai
10	Ve-65-2	*V. dahurica* Steven × *V. pyrethrina* Nakai
11	Ve-75	*V. kiusiana* var. glabrifolia × *V. spicata* f. nana ‘Blauteppich’
12	Ve-77	*V. kiusiana* var. glabrifolia × *V. spicata* f. nana ‘Blauteppich’
13	Ve-86	*V. kiusiana* var. glabrifolia × *V. dahurica* Steven
14	Ve-87	*V. kiusiana* var. glabrifolia × *V. pusanensis* Y. N. Lee
15	Ve-88	*V. kiusiana* var. glabrifolia × *V. pusanensis* Y. N. Lee
16	Ve-91	*V. nakaiana* Ohwi × *V. spicata* f. nana ‘Blauteppich’
17	Ve-109	*V.* ‘Veronica Blue’ × *V. kiusiana* var. glabrifolia
18	Ve-131	*V. spicata* f. nana ‘Blauteppich’ × *V. pyrethrina* Nakai
19	Ve-132	*V. spicata* f. nana ‘Blauteppich’ × *V. pyrethrina* Nakai
20	Ve-153	*V. spicata* ‘Ulster Blue Dwarf’ × *V. kiusiana* var. glabrifolia

## Data Availability

The data presented in this study are available within the article.

## References

[1] Trask B.J. (2002). Human cytogenetics: 46 chromosomes, 46 years and counting. Nat. Rev. Genet..

[2] Speicher M.R., Carter N.P. (2005). The new cytogenetics: Blurring the boundaries with molecular biology. Nat. Rev. Genet..

[3] Hwang Y.J., Cabahug R.A., Mancia F.H., Lim K.B. (2020). Molecular cytogenetics and its application to major flowering ornamental crops. Hort. Environ. Biotech..

[4] Hwang Y.J., Song C.M., Kwon M.K., Kim S.T., Kim W.H., Han Y.Y., Han T.H., Lim K.B. (2010). An increment of crossing efficiency with consideration of pollen viability analysis in rose. Flower Res. J..

[5] Wang Q., Wang J., Zhang Y., Zhang Y., Xu S., Lu Y. (2015). The application of fluorescence in situ hybridization in different ploidy level cross-breeding of lily. PLoS ONE.

[6] Albach D.C., Martínez-Ortega M.M., Delgado L., Weiss-Schneeweiss H., Özgökce F., Fischer M.A. (2008). Chromosome numbers in Veroniceae (Plantaginaceae): Review and several new counts. Ann. Mo. Bot. Gar..

[7] Albach D.C., Meudt H.M., Oxelman B. (2005). Piecing together the “new” Plantaginaceae. Am. J. Bot..

[8] Oh H.Y., Shin U.S., Song S.J., Kim J.H., Kim S.Y., Suh G.U. (2019). Growth and flowering characteristics of 20 *Veronica* species. Flower Res. J..

[9] Albach D.C., Briggs B.G. (2012). Phylogenetic analysis of Australian species of *Veronica* (*V.* section *Labiatoides*; Plantaginaceae). Aust. Syst. Bot..

[10] Albach D.C., Chase M.W. (2004). Incongruence in Veroniceae (Plantaginaceae): Evidence from two plastid and a nuclear ribosomal DNA region. Mol. Phylogenet. Evol..

[11] Song C.Y., Moon J.Y., Kim S.Y. (2020). Growth and flowering characteristics of *Veronica* native to Korea and their crossings. Acta Hortic..

[12] Albach D.C., Greilhuber J. (2004). Genome size variation and evolution in *Veronica*. Ann. Bot..

[13] Müller K., Albach D.C. (2010). Evolutionary rates in *Veronica* L. (Plantaginaceae): Disentangling the influence of life history and breeding system. J. Mol. Evol..

[14] Sharma N., Koul P., Koul A.K. (1993). Pollination biology of some species of genus *Plantago* L.. Bot. J. Linn. Soc..

[15] Britten E.J. (1960). A proposed classification of screening methods for plant breeding programs. Euphytica.

[16] Väinölä A. (2000). Polyploidization and early screening of *Rhododendron* hybrids. Euphytica.

[17] Stetter M.G., Zeitler L., Steinhaus A., Kroener K., Biljecki M., Schmid K.J. (2016). Crossing methods and cultivation conditions for rapid production of segregating populations in three grain amaranth species. Front. Plant Sci..

[18] Lakshmanan P.S., Van Laere K., Eeckhaut T., Van Huylenbroeck J., Van Bockstaele E., Khrustaleva L. (2015). Karyotype analysis and visualization of 45S rRNA genes using fluorescence in situ hybridization in aroids (Araceae). Comp. Cytogenet..

[19] Van Laere K., Van Huylenbroeck J., Van Bockstaele E. (2008). Karyotype analysis and physical mapping of 45S rRNA genes in *Hydrangea* species by fluorescence in situ hybridization. Plant Breed..

[20] Schubert I., Fransz P.F., Fuchs J., de Jong J.H. (2001). Chromosome painting in plants. Methods Cell Sci..

[21] Leitch I.J., Heslop-Harrison J.S. (1992). Physical mapping of the 18S–5.8 S–26S rRNA genes in barley by in situ hybridization. Genome.

[22] Heslop-Harrison J.S. (2000). Comparative genome organization in plants: From sequence and markers to chromatin and chromosomes. Plant Cell.

[23] Ha M.K.T.T., Sevilleno S.S., Cabahug R.A.M. (2022). Cytogenetic analysis and genome size estimation of Korean native Veronica taxa. Flower Res. J..

[24] Garcia S., Kovařík A., Leitch A.R., Garnatje T. (2017). Cytogenetic features of rRNA genes across land plants: Analysis of the Plant rDNA database. Plant J..

[25] Ramzan F., Kim H.T., Shim K.K., Younis A., Choi Y.H., Hwang Y.J., Lim K.B. (2018). Evaluation of F1 and BC1 hybrids of *Lilium lancifolium* × Asiatic hybrid ‘Chianti’ by morphological analysis and fluorescence in situ hybridization. J. Hortic. Sci. Biotechnol..

[26] Li X., Zheng B., Xu W., Ma X., Wang S., Qian M., Wu H. (2022). Identification of F1 hybrid progenies in mango based on Fluorescent SSR markers. Horticulturae.

[27] Sari H., Eker T., Sari D., Aksoy M., Bakır M., Dogdu V., Toker C., Canci H. (2023). The Fastest and Most Reliable Identification of True Hybrids in the Genus *Pisum* L.. Life.

[28] Farshadfar M. (2020). Chromosome location of ISSR markers and genes controlling seed germination under drought stress in wheat-barley disomic addition lines. Cell. Mol. Biol..

[29] Guerra M. (2008). Chromosome numbers in plant cytotaxonomy: Concepts and implications. Cytogenet. Genome Res..

[30] Sun W., Sun H., Li Z. (2019). Chromosome data mining and its application in plant diversity research. Plant Sci. J..

[31] Devi J., Ko J.M., Seo B.B. (2005). FISH and GISH: Modern cytogenetic techniques. Indian J. Biotechnol..

[32] Waminal N.E., Kim H.H. (2012). Dual-color FISH karyotype and rDNA distribution analyses on four Cucurbitaceae species. Hortic. Environ. Biotech..

[33] Belandres H.R., Waminal N.E., Hwang Y.J., Park B.S., Lee S.S., Huh J.H., Kim H.H. (2015). FISH karyotype and GISH meiotic pairing analyses of a stable intergeneric hybrid *xBrassicoraphanus* line BB#5. Korean J. Hortic. Sci. Technol..

[34] Younis A., Ramzan F., Hwang Y.J., Lim K.B. (2015). FISH and GISH: Molecular cytogenetic tools and their applications in ornamental plants. Plant Cell Rep..

[35] Marasek A., Hasterok R., Wiejacha K., Orlikowska T. (2004). Determination by GISH and FISH of hybrid status in *Lilium*. Hereditas.

[36] Su D., Chen L., Sun J., Zhang L., Gao R., Li Q., Han Y., Li Z. (2020). Comparative chromosomal localization of 45S and 5S rDNA sites in 76 purple-fleshed sweet potato cultivars. Plants.

[37] Silva G.S., Souza M.M., de Melo C.A., Urdampilleta J.D., Forni-Martins E.R. (2018). Identification and characterization of karyotype in *Passiflora* hybrids using FISH and GISH. BMC Genet..

[38] Li D., Li T., Wu Y., Zhang X., Zhu W., Wang Y., Zeng J., Xu L., Fan X., Sha L. (2018). FISH-based markers enable identification of chromosomes derived from tetraploid *Thinopyrum elongatum* in hybrid lines. Front. Plant Sci..

[39] Tucker S., Vitins A., Pikaard C.S. (2010). Nucleolar dominance and ribosomal RNA gene silencing. Cur. Opin. Cell Biol..

[40] Matyasek R., Dobesova E., Huska D., Ježková I., Soltis P.S., Soltis D.E., Kovařík A. (2016). Interpopulation hybridization generates meiotically stable rDNA epigenetic variants in allotetraploid *Tragopogon mirus*. Plant J..

[41] Volkov R.A., Panchuk I.I., Borisjuk N.V. (2017). Evolutional dynamics of 45S and 5S ribosomal DNA in ancient allohexaploid *Atropa belladonna*. BMC Plant Biol..

[42] Nishida S., Tamakoshi N., Takakura K.I., Watanabe Y., Kanaoka M.M. (2024). Reproductive interference between alien species in *Veronica*. J. Plant Res..

[43] Wilson G.B., Houston L., Whittington W.J., Humphries R.N. (2000). *Veronica spicata* L. ssp. *spicata* and ssp. *hybrida* (L.) Gaudin (*Pseudolysimachium spicatum* (L.) Opiz). J. Ecol..

[44] Yu D., Gu X., Zhang S., Dong S., Miao H., Gebretsadik K., Bo K. (2021). Molecular basis of heterosis and related breeding strategies reveal its importance in vegetable breeding. Hortic. Res..

[45] Gros-Louis M.C., Bousquet J., Pâques L.E., Isabel N. (2005). Species-diagnostic markers in Larix spp. based on RAPDs and nuclear, cpDNA, and mtDNA gene sequences, and their phylogenetic implications. Tree Genet. Genomes.

[46] Desel C., Jansen R., Dedong G., Schmidt T. (2002). Painting of parental chromatin in beta hybrids by multi-colour fluorescent in situ hybridization. Ann. Bot..

[47] Markova M., Lengerova M., Ziuvova J., Janousek B., Vyskot B. (2006). Karyological analysis of an interspecific hybrid between the dioecious *Silene latifolia* and the hermaphroditic *Silene viscose*. Genome.

[48] Lim K.B., De Jong H., Yang T.J., Park J.Y., Kwon S.J., Kim J.S., Lim M.H., Kim J.A., Jin M., Jin Y.-M. (2005). Characterization of rDNAs and tandem repeats in the heterochromatin of *Brassica rapa*. Mol. Cells.

[49] Waminal N.E., Pellerin R.J., Kim N.S., Jayakodi M., Park J.Y., Yang T.J., Kim H.H. (2018). Rapid and efficient FISH using pre-labeled oligomer probes. Sci. Rep..

